# Effort required to finish shotgun-generated genome sequences differs significantly among vertebrates

**DOI:** 10.1186/1471-2164-11-21

**Published:** 2010-01-11

**Authors:** Robert W Blakesley, Nancy F Hansen, Jyoti Gupta, Jennifer C McDowell, Baishali Maskeri, Beatrice B Barnabas, Shelise Y Brooks, Holly Coleman, Payam Haghighi, Shi-Ling Ho, Karen Schandler, Sirintorn Stantripop, Jennifer L Vogt, Pamela J Thomas, NISC Comparative Sequencing Program, Gerard G Bouffard, Eric D Green

**Affiliations:** 1 NIH Intramural Sequencing Center (NISC), National Human Genome Research Institute, National Institutes of Health, Bethesda, MD 20892, USA; 2 Genome Technology Branch, National Human Genome Research Institute, National Institutes of Health, Bethesda, MD 20892, USA

## Abstract

**Background:**

The approaches for shotgun-based sequencing of vertebrate genomes are now well-established, and have resulted in the generation of numerous draft whole-genome sequence assemblies. In contrast, the process of refining those assemblies to improve contiguity and increase accuracy (known as 'sequence finishing') remains tedious, labor-intensive, and expensive. As a result, the vast majority of vertebrate genome sequences generated to date remain at a draft stage.

**Results:**

To date, our genome sequencing efforts have focused on comparative studies of targeted genomic regions, requiring sequence finishing of large blocks of orthologous sequence (average size 0.5-2 Mb) from various subsets of 75 vertebrates. This experience has provided a unique opportunity to compare the relative effort required to finish shotgun-generated genome sequence assemblies from different species, which we report here. Importantly, we found that the sequence assemblies generated for the same orthologous regions from various vertebrates show substantial variation with respect to misassemblies and, in particular, the frequency and characteristics of sequence gaps. As a consequence, the work required to finish different species' sequences varied greatly. Application of the same standardized methods for finishing provided a novel opportunity to "assay" characteristics of genome sequences among many vertebrate species. It is important to note that many of the problems we have encountered during sequence finishing reflect unique architectural features of a particular vertebrate's genome, which in some cases may have important functional and/or evolutionary implications. Finally, based on our analyses, we have been able to improve our procedures to overcome some of these problems and to increase the overall efficiency of the sequence-finishing process, although significant challenges still remain.

**Conclusion:**

Our findings have important implications for the eventual finishing of the draft whole-genome sequences that have now been generated for a large number of vertebrates.

## Background

Currently, the standard and most efficient approach for the de novo generation of sequence from complex genomes (e.g., those of vertebrates) is 'shotgun sequencing' [1,2], although new technologies are emerging. In the initial 'shotgun' phase, large numbers of overlapping sequence reads are obtained at random from a large-insert clone or a whole genome, yielding highly redundant sequence data that typically represent the vast majority of the starting DNA. In the second 'finishing' (or refinement) phase, the resulting draft sequence assemblies are analyzed and refined, often with additional sequence data generated to improve continuity and accuracy. Through considerable effort, the sequence can be refined to near perfection--something sought by the Human Genome Project in generating a finished sequence of the human genome [3,4]. However, the generation of such high-quality genomic sequence requires extensive sequence-finishing efforts, which involve a low-throughput, craftsman-like process carried out by highly-skilled personnel performing both customized computational and experimental procedures. Alternatively, genomic sequence assemblies can be partially refined to less rigorous quality standards at a fraction of the cost and effort, with the resulting sequences still being extremely valuable for numerous applications, including comparative analyses [5-7]. Anecdotal experiences to date in sequencing vertebrate genomes have clearly revealed that each species' genome presents its own characteristic set of challenges. Meanwhile, draft (but not finished) whole-genome sequences have now been generated for a large number of mammalian and other vertebrate species [8,9], without clear plans for finishing these genome sequences.

The NISC Comparative Sequencing Program [10] has been extensively involved in inter-species sequence comparisons. Specifically, we have generated sequence data from many vertebrates in parallel, focusing our studies on targeted genomic regions (as opposed entire genomes). This work has involved isolating, mapping, and sequencing orthologous bacterial artificial chromosome (BAC) clones from the same well-defined 0.5-2 Mb regions [6,11] of multiple vertebrate genomes and systematically comparing the resulting sequences [6,7,12-20]. Our collective efforts to date have resulted in the generation of sequence for over 11,000 BACs isolated from 75 vertebrate species. The largest fraction of these data was produced as part of the ENCODE project [21].

In generating these BAC sequences, we have paid considerable attention to the sequence-finishing process. We routinely refine all BAC-derived sequence assemblies to produce 'comparative-grade' finished sequence [5], the quality specifications of which were specifically designed for inter-species sequence comparisons. Comparative-grade finished sequence is produced by establishing the order and orientation of sequence contigs greater than 2 kb in size, and then independently verifying the resulting sequence contig map by auxiliary data. Our studies have shown that the quality of comparative-grade finished sequence is very high, with residual gaps and errors mostly residing within repetitive sequences [5]. To date, a subset of our sequenced BACs (>925) have also been finished to the near-perfect standards established for sequencing the human genome [4], which we refer to as 'human-grade' finished sequence.

Our efforts in sequencing the same orthologous genomic regions in many different species and in producing two 'grades' of finished sequence for a large subset of BACs provided us a unique opportunity to examine results from application of the same standardized process to a variety of genome sequences. Here, we report a series of studies that reveal substantial differences in the effort required to finish shotgun-generated genome sequences from different vertebrates, reflecting significant architectural differences between orthologous regions of these vertebrate genomes.

## Results

### Sequence data sets

The nature of our comparative genome mapping and sequencing program [6,11] has required us to routinely sequence the DNA from many vertebrates. Our data and the anecdotal experiences of others have consistently suggested marked differences among species with respect to the effort required to refine (i.e., finish) shotgun-generated sequence assemblies, even for the same orthologous genomic region. To investigate these differences in a more systematic fashion, we generated and analyzed two comparable sequence data sets.

The first data set (Table 1) consists of the human-grade finished sequences generated for 541 BACs from 38 vertebrates, with all sequences orthologous to a 1.9-Mb genomic region (in human) encompassing the cystic fibrosis transmembrane conductance regulator gene (*CFTR*); this genomic segment corresponds to ENCODE pilot project region ENm001 [22,23]. The number of sequenced BACs and the total amount of sequence in the ENm001-derived multiple-BAC sequence assembly generated for each vertebrate are provided in Table 1. A species' sequence was included in this data set if PipMaker-derived alignments [24] of that species' BAC sequences together covered at least 75% of the human ENm001 reference sequence. Importantly, comparative-grade finished sequence was also available for all 541 sequenced BACs in this first data set.

**Table 1:** Human-grade finished BAC sequences from ENm001.

Common name	Taxonic name	Total BACs	Total Mb
Armadillo	*Dasypus novemcinctus*	25	2.63
Baboon	*Papio anubis*	15	2.26
Black Lemur	*Eulemur macaco macaco*	8	1.41
Cat	*Felis catus*	20	2.11
Chicken	*Gallus gallus*	7	0.84
Chimpanzee	*Pan troglodytes*	13	1.73
Colobus Monkey	*Colobus guereza*	14	2.12
Cow	*Bos taurus*	15	2.34
Dog	*Canis familiaris*	9	1.34
Dusky Titi	*Callicebus moloch*	17	2.29
Elephant	*Loxodonta africana*	24	2.25
Galago	*Otolemur garnettii*	13	2.17
Gibbon	*Nomascus leucogenys leucogenys*	16	2.43
Gorilla	*Gorilla gorilla gorilla*	11	1.94
Ground Squirrel	*Spermophilus tridecemlineatus*	16	1.91
Guinea Pig	*Cavia porcellus*	15	2.01
Hedgehog	*Atelerix albiventris*	25	2.86
Horse	*Equus caballus*	14	2.13
Horseshoe Bat	*Rhinolophus ferrumequinum*	16	1.98
Little Brown Bat	*Myotis lucifugus*	15	2.16
Macaque	*Macaca mulatta*	14	1.79
Marmoset	*Callithrix jacchus*	13	2.13
Mouse Lemur	*Microcebus murinus*	10	1.76
Orangutan	*Pongo pygmaeus*	13	2.02
Owl Monkey	*Aotus nancymaae*	18	2.39
Pig	*Sus scrofa*	10	1.50
Platypus	*Ornithorhynchus anatinus*	16	1.68
Rabbit	*Oryctolagus cuniculus*	17	2.32
Rat	*Rattus norvegicus*	17	2.16
Ring-tailed Lemur	*Lemur catta*	10	1.51
Sheep	*Ovis aries*	15	2.18
Shrew	*Sorex araneus*	19	2.07
Squirrel Monkey	*Saimiri boliviensis boliviensis*	15	2.38
Tenrec	*Echinops telfairi*	12	1.92
Tetraodon	*Tetraodon nigroviridis*	3	0.32
Torafugu	*Takifugu rubripes*	2	0.17
Vervet Monkey	*Chlorocebus aethiops*	13	1.93
Wallaby	*Macropus eugenii*	16	2.05

Totals		541	73.20

The total number of BACs (Total BACs) and the amount of non-redundant human-grade finished sequence generated (Total Mb) for each species within genomic region ENm001 are shown.

The second data set consists of the comparative-grade finished sequences generated for 2,031 BACs from 21 vertebrate species (Table 2), with each sequence orthologous to one of 14 ENCODE pilot project regions (Table 3). The number of sequenced BACs and the total amount of sequence in the individual BAC clone sequences generated for each species (Table 2) or ENCODE region (Table 3) are listed. To be included in this data set, more than 75% of each of the ENCODE pilot project regions (Table 3) needed to be covered by PipMaker-derived alignments of comparative-grade finished sequence from that species (Table 2). Most of the BAC sequences in this second data set have not been refined to human-grade finished sequence. Because of insufficient sequence coverage, BAC sequences were not included in this second data set from three other ENCODE pilot project regions (ENr231, ENr232, and ENr333) that have unusual characteristics and were thus analyzed for illustrative purposes (e.g., see below); in these cases, the number of sequenced BACs (and total Mb of individual BAC clone sequences) for ENr231, EN232, and ENr333 were 64 (10.3 Mb), 51 (8.0 Mb), and 62 (10.0 Mb), respectively.

**Table 2:** Comparative-grade finished BAC sequences listed by species.

Species	Total BACs	Total Mb
Armadillo	137	18.8
Baboon	110	19.2
Cat	105	14.6
Colobus Monkey	78	16.2
Dusky Titi	88	15.3
Elephant	138	18.8
Galago	99	18.5
Gibbon	83	14.9
Ground Squirrel	89	13.4
Guinea Pig	93	15.6
Hedgehog	130	19.4
Horseshoe Bat	95	15.5
Marmoset	97	19.0
Mouse Lemur	60	13.8
Owl Monkey	88	15.3
Platypus	76	10.8
Rabbit	95	17.4
Shrew	115	15.7
Squirrel Monkey	73	14.6
Tenrec	95	17.1
Vervet Monkey	87	14.5

Totals	2,031	338.5

The total number of BACs (Total BACs) and the amount of comparative-grade finished sequence generated (Total Mb) for each species are shown.

**Table 3:** Comparative-grade finished BAC sequences listed by ENCODE region.

ENCODE region	Size in human (kb)	Total BACs	Total Mb
ENm001	1,877	357	58.9
ENm003	500	91	15.3
ENm005	1,696	256	42.6
ENm010	500	96	16.1
ENm012	1,000	179	29.4
ENm013	1,114	184	30.3
ENm014	1,163	214	35.4
ENr111	500	84	14.4
ENr211	500	89	15.2
ENr213	500	90	15.4
ENr221	500	100	16.5
ENr222	500	98	16.5
ENr312	500	94	15.7
ENr323	500	99	16.9

Totals	14,850	2,031	338.5

The total number of BACs (Total BACs) and the amount of comparative-grade finished sequence generated (Total Mb) for each genomic region are shown.

### Misassembled sequence

In shotgun sequencing, once a sufficient number of sequence reads are collected based on the size of the starting template (e.g., a BAC), the sequence is assembled computationally (such as with the program Phrap [25,26]). Even though the assembly program is operated under conditions we have optimized for our BAC sequences, occasionally an incorrect alignment occurs with closely related but distinct reads, resulting in a misassembled and incorrect consensus sequence. Misassemblies can result from various causes, such as the presence of multiple copies of closely-related repetitive sequences that inappropriately collapse onto one or a few regions. Often, misassemblies incorrectly portray non-adjacent segments as being contiguous. Such problems require manual correction, otherwise the sequence would be of marginal utility (e.g., for accurately identifying genes or characterizing the long-range organization of a genomic region). With the aid of sequence-editing programs, an experienced technician systematically reviews and sorts the misassembled reads, eventually correcting problems and allowing a valid consensus sequence to emerge. However, this manual intervention can be one of the most labor-intensive and costly components of the sequence-finishing process. Details of our sequence-finishing process are provided in the Supplementary Materials associated with Blakesley *et al*. [5].

We have consistently found that certain genomic regions are more prone to sequence misassemblies than others, regardless of the species being studied. For example, considerable effort was required to resolve misassembled sequences for more than 20% of all BACs from ENCODE pilot project regions ENr231 and ENr333; for the other ENCODE regions, less than 5% of the BACs needed such attention. Similarly, certain species stand out as being more prone to sequence misassemblies, regardless of the genomic region being sequenced. For example, baboon BAC sequences typically contain an unusually high frequency of long (2- to 43- kb) inverted and tandem repeats of >90% identity that greatly confound standard sequence-assembly routines. As another example, assembled hedgehog sequences often contained non-contiguous segments that had been incorrectly joined by Phrap. In these cases, more extensive manual effort was required to untangle the misassemblies.

We investigated alternative sequence-assembly programs to Phrap that provide 'read-pair awareness' as a means of reducing the amount of manual correction of the initial sequence assembly required. Of the many programs evaluated, rPhrap (Geospiza) [27] and Autosort (NFH, unpublished) showed the greatest promise. We thus tested these two sequence-assembly programs by having them assemble the sequence reads generated from 102 BACs that had previously been found to be associated with Phrap-induced sequence misassemblies. Improved sequence assemblies were generated for 68 of these BACs (with complete resolution for 29 BACs and reduction of minor misassemblies for 39 BACs). Direct comparison of the performance of each program revealed that one generally showed more improvement than the other for any particular BAC, but Autosort overall yielded improved assemblies for twice as many BACs as rPhrap. While these findings represent a positive development, it is important to note that these alternative sequence-assembly programs are actually less effective than Phrap for assembling average BAC sequences, due primarily to a great increase in the number of contigs and occasional generation of new misassemblies.

We also tested whether improved sequence assemblies could be generated by separately altering certain Phrap parameters. The shotgun sequence reads for ten of the above BACs were assembled using four different sets of Phrap parameters; each resulting assembly was then analyzed for features we have found to serve as a measure of the manual effort required for sequence finishing (specifically, the total number of contigs, misjoined sequences, uncaptured gaps, contigs which can be manually joined, and groups of reads to remove from contigs). Compared to the assemblies generated with our optimized Phrap parameters, those generated using the 'repeat stringency' set at 99 or 'shatter greedy' were inferior with respect to the above measures, and would have required substantial additional effort to complete the finishing process. Assemblies generated using 'revise greedy' or a 'forcelevel' of 3 (instead of 0) were found to be similar to our standard optimized Phrap assemblies. Based on analyses such as this, we have adopted a routine in which shotgun sequence reads are first assembled with Phrap using our optimized parameters; if problematic misassemblies are noted, the reads are then reassembled with rPhrap and separately with Autosort. The best of the three assemblies is then selected as a better starting point for further refinement, including manual resolution of more complex misassemblies.

### Frequency of gaps

When inspecting a sequence assembly, one of the first features to assess is whether the consensus sequence is in a single piece or multiple pieces (i.e., contigs). An assembly consisting of more than one contig indicates that the sequencing procedure failed to generate sufficient sequence reads from the DNA residing between contigs (i.e., gaps). The number of sequence gaps is a first indicator of the amount of effort that will be required to finish an assembled sequence. Early in any sequence-finishing process, each contig must be manually inspected, looking for internal regions of low quality or inappropriately joined segments. As might be anticipated, contig ends typically contain poor-quality sequence, either due to individual sequence reads extending well beyond the average quality length or due to chimeric reads. The amount of effort required to manually inspect and refine contig ends is directly proportional to their number (each gap is associated with two flanking contig ends).

The impact of contig number is most pronounced during the steps involved in establishing contig order and orientation, a key part of the finishing process. One approach involves comparing in silico- and laboratory-generated restriction enzyme digestion-based fingerprints of a BAC. When the assembled sequence is distributed among numerous contigs, in silico-deduced restriction fragments become artificially broken, making it is nearly impossible to derive an accurate restriction enzyme digestion-based fingerprint from the many combinatorial possibilities. A second approach for ordering and orienting contigs involves aligning each contig to an orthologous reference sequence. Once again, interpreting the alignments of multiple small contigs in an assembled sequence is far more difficult than just a few large contigs (especially for sequences from species more distantly related to the reference sequence).

For these reasons, the number of gaps in an assembled sequence is a critical parameter for the sequence-finishing process. We analyzed the first data set for the presence of gaps in the comparative-grade finished sequence of the 541 BACs. Specifically, for each of the 38 species listed in Table 1, we aligned the comparative-grade finished sequence of each BAC to the corresponding human-grade finished sequence for that species using the program Cross Match [28]. The resulting alignments were manually examined and corrected when contigs in the comparative-grade finished sequence had an ambiguous endpoint and/or order in the alignment. From the final alignments, we then catalogued the location, size, and sequence composition of all gaps in the comparative-grade finished sequence; further, the number of shotgun-library subclones spanning each gap (as detected by establishing the locations of all sequence reads in the human-grade finished sequence) was also determined. Across the 73.2 Mb of total sequence in the first data set (Table 1), we identified 1,528 gaps, corresponding to roughly 23 gaps per Mb. Notably, we found an identical frequency of gaps in an earlier study that involved analyzing the comparative-grade finished sequence of 116 vertebrate BACs [5]. Those 1,528 gaps together account for 1.1 Mb (or 1.5%) of the total sequence, and range in size from 1 bp to greater than 15 kb, with a median size of 250 bp; roughly a third (506) of the gaps are 500 bp or larger.

**Figure 1:**
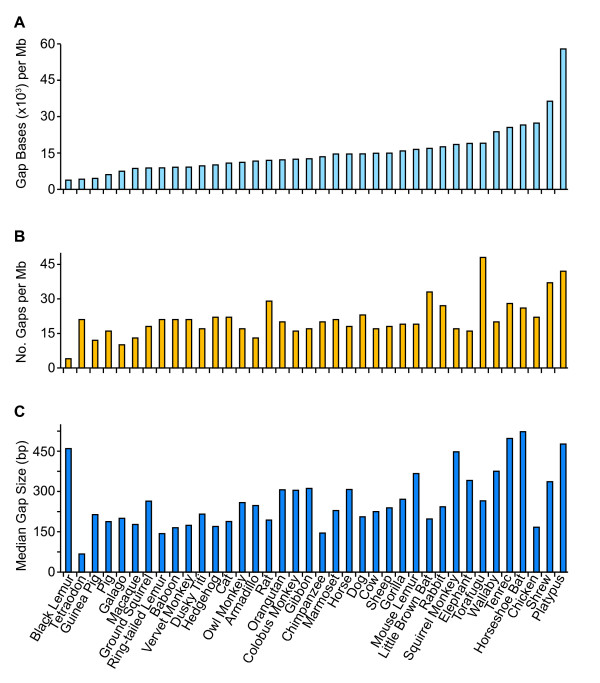
**Gaps in comparative-grade finished BAC sequences from ENm001**. The human- and comparative-grade finished sequences of the 541 BACs summarized in Table 1 were compared, and the gaps detected in the comparative-grade finished sequence were analyzed. Indicated for each species are the total bases within gaps per Mb of human-grade finished sequence (**A**), the number of gaps per Mb of human-grade finished sequence (**B**), and the median size of the gaps in base pairs (**C**).

Examination of the simple aggregate statistics above fails to reveal the striking differences between individual species with respect to gaps in their assembled genome sequences. For example, the amount of sequence residing within gaps varied more than 15-fold among species (Figure 1A). For species like black lemur, tetraodon, and guinea pig, gap sizes are relatively small (~ 4 kb per Mb), whereas the gaps in shrew and platypus sequence correspond to an average of 36 and 58 kb per Mb, respectively. In the case of gap frequency, variation is less pronounced, i.e., less than fourfold for all but three of the 38 species; in fact, gap frequency ranged from 17 to 23 per Mb for over half of the species, near the overall median value of 20 gaps per Mb (Figure 1B). At the extremes for gap frequency are: (1) at the low end, black lemur and galago, with an average of 4 and 10 gaps per Mb, respectively; and (2) at the high end, platypus and torafugu, with an average of 42 and 48 gaps per Mb, respectively. A third characteristic, median gap size (Figure 1C), varies dramatically--from 67 bp for tetraodon to 523 bp for horseshoe bat; platypus is again at the high end, with a median gap size of 477 bp.

An overall comparison of frequency and size of sequence gaps indicates that some species' sequences (e.g., guinea pig and galago) have below average values; the finishing of these sequences is therefore more straightforward. Chicken sequences are intermediate, with a high frequency of relatively small gaps. Finally, at the extreme of difficulty are platypus sequences, which have substantially more gaps of larger size (compared to the overall averages).

### Characteristics of sequences in gaps

**Figure 2:**
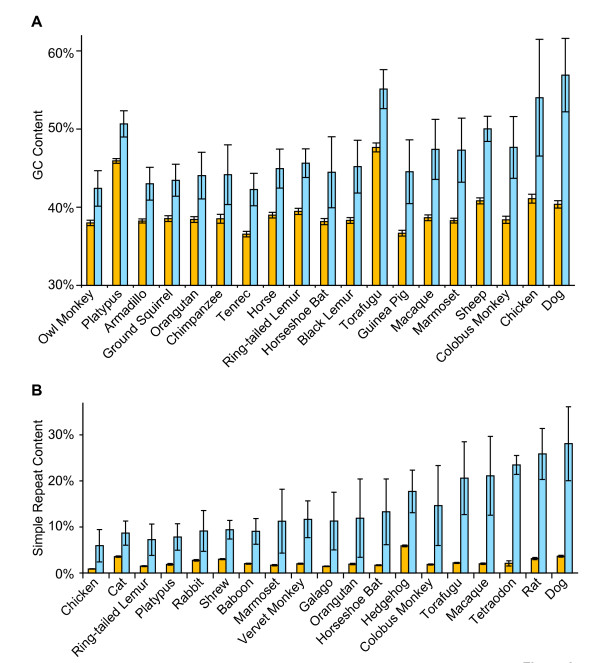
**Characteristics of gaps in comparative-grade finished BAC sequences from ENm001**. Sequences within the gaps (blue bars) summarized in Figure 1 were analyzed with respect to GC (**A**) and simple repeat (**B**) content; similar analyses were performed for the entire human-grade finished BAC sequences (orange bars). Results for the 19 species with the greatest differences (i.e., gap sequences vs. total BAC sequences) for each analysis are shown. Each error bar represents the 95% confidence interval.

The characteristics of the sequence within and immediately adjacent to a gap greatly influence the difficulty associated with filling that gap during sequence finishing. We thus examined the GC and repeat content of the sequence residing within gaps in the first data set, comparing those values to the overall averages for the total sequence generated for each species. The results for the 19 species with the greatest differences between gap sequence and total sequence are shown in Figure 2.

For many species, the GC content of the gap sequence is only marginally different from the GC content of the total sequence (Figure 2A). For some species where large differences are noted (e.g., guinea pig, marmoset, and chicken), we have not found human-grade sequence finishing to be particularly difficult; the application of standard finishing strategies involving alternate sequencing reaction chemistry (e.g., 80:20 BigDye:dGTP BigDye or dGTP BigDye supplemented with SeqRxA) generally permits the generation of the missing sequence with minimal effort. However, this is not the case for other species. For example, the gaps in dog sequence average 57% GC content, considerably higher than the 40% GC content of the total sequence. One particular dog BAC sequence [GenBank:AC090445] contains a stretch of 2,100 bp with a GC content of 83%; an embedded gap of ~ 250 bp (85% GC content overall) was refractory to most routine gap-filling efforts and ultimately required an extraordinary finishing effort to close. Several other dog BAC sequences contain at least one gap that presented similar challenges during finishing. Interestingly, such high GC-content regions in dog sequence gaps often reside near CpG islands.

Previously, we found that sequence within gaps is disproportionately more repetitive than non-gap sequence; specifically, total repetitive sequences identified by the program RepeatMasker [29] account for roughly 50% of gap bases in comparative-grade finished sequence, while the subset of simple repeat sequences (predominantly homopolymer, as well as di-, tri, and tetranucleotide repeats) account for 3.7% of gap bases [5]. Further, it is well-established that these simple repeat sequences present major challenges during sequence finishing (e.g., determining the exact length of a dinucleotide repeat). We thus compared the simple repeat content of gap sequences for the species in the first data set (Figure 2B). In aggregate for all species, 2% of the total sequence corresponds to simple repeats, whereas 10% of the sequence in captured gaps corresponds to simple repeats. Thus, simple repeats are generally enriched within captured gaps. The considerable variability seen among species with respect to this enrichment (Figure 2B) is even more dramatic. At the extreme, dog has an unusually high amount of simple repeats in its gap sequence (28%); by comparison, in 26 of 37 other species, this value is less than 10%. Table 4 details the repeat content within all the sequences of our first data set (taken as a whole) as well as that in captured and uncaptured gaps. In addition to again noting the significant enrichment of simple repeats within captured gaps, these data reveal that long interspersed repeats (LINES) are significantly enriched in uncaptured gaps.

**Table 4:** Repeat content of total sequences and gap sequences.

Repeat Type	Total Sequence	Total Gaps	Captured Gaps	Uncaptured Gaps
All Repeats	36.9 (0.4)	48.9 (1.4)	45.1 (1.7)	49.9 (1.7)
Simple	1.6 (0.0)	4.6 (0.7)	10.3 (1.0)	2.9 (0.5)
LTR	4.7 (0.1)	5.6 (0.7)	2.8 (0.5)	6.4 (0.8)
SINE	9.3 (0.2)	8.8 (0.7)	9.6 (1.0)	8.6 (0.5)
LINE	18.6 (0.3)	27.9 (1.3)	20.6 (1.6)	29.9 (1.8)
DNA	2.7 (0.0)	2.0 (0.2)	1.8 (0.4)	2.1 (0.4)

Sequences of the 541 BACs (Table 1) were analyzed in aggregate by RepeatMasker. The percentages of bases represented by the indicated types of sequence repeats are reported for the total sequence, total gaps, captured gaps, and uncaptured gaps. Numbers in parenthesis are the one sigma error values.

### Captured versus uncaptured gaps

An early step in sequence finishing involves ordering and orienting contigs within the initial sequence assembly. This process extensively utilizes information about the paired forward- and reverse-primed sequence reads generated from each plasmid template during the shotgun phase. Two contig ends are considered adjacent to each other if two or more sequence reads in one contig connect to their plasmid 'mates' in the other contig; in such cases, the intervening gap is considered 'captured' by the spanning subclones. Furthermore, the calculated minimum distance between read pairs should not greatly exceed the average insert size of the subclone library. Sequence gaps not spanned by at least two appropriately spaced sequence read-pairs are deemed 'uncaptured' and add no spatial information to the sequence contig map. An assembly containing several uncaptured gaps can thus be quite challenging to finish, with other data required to deduce contig order and orientation; substantial additional effort is frequently involved during the finishing process, particularly in cases where the auxiliary data are weak or limited. Furthermore, generating the sequence of regions within captured gaps (e.g., during human-grade sequence finishing) is most readily achieved by sequencing the spanning plasmid subclones; in contrast, generating the sequence of regions within uncaptured gaps requires sequencing alternative templates (e.g., PCR products or purified BAC DNA), which is more costly and labor-intensive.

**Figure 3:**
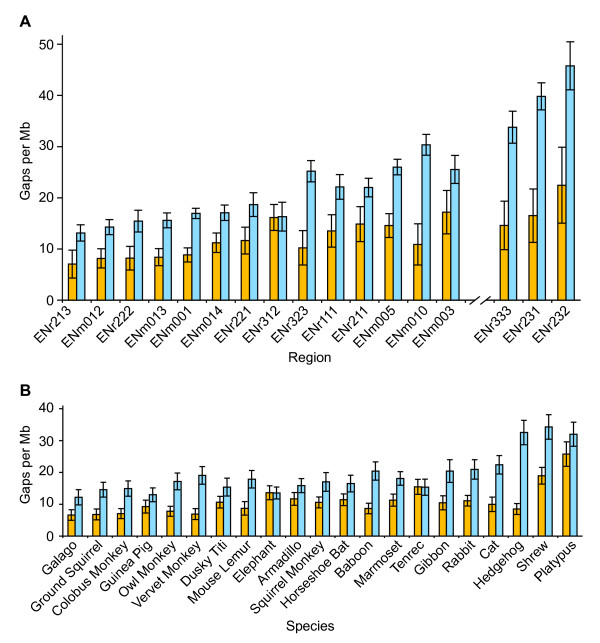
**Gaps in comparative-grade finished BAC sequences from multiple genomic regions**. The comparative-grade finished sequences of the 2,031 BACs summarized in Tables 2 and 3 were analyzed for the presence of uncaptured (orange bar) and captured (blue bar) gaps (see text for details). The numbers of captured and uncaptured gaps per Mb averaged across all BACs from each indicated genomic region (**A**) or species (**B**) are indicated. Each error bar represents the 95% confidence interval. In **A**, the data for three additional ENCODE pilot project regions (ENr231, ENr232, and ENr333) are shown for comparison because of the notably high frequency of gaps in their sequences; however, there were not sufficient numbers of sequenced BACs from these regions to qualify for inclusion in the second data set (see text for details).

We analyzed the comparative-grade finished sequence generated for the 2,031 BACs in the second data set for the presence of captured and uncaptured gaps. In aggregate, these sequences contain roughly 30 gaps per Mb, with approximately two-thirds of these gaps being captured. This result is generally expected for shotgun sequence assemblies with at least eightfold sequence redundancy. However, the characteristics of the sequence gaps vary among genomic regions and among species (Figure 3). For example, the average total number of gaps within the sequences generated for these 14 ENCODE pilot project regions ranges from 20 per Mb (ENr213) to 43 per Mb (ENm003), while the fraction of uncaptured gaps varies from 26% (ENm010) to 50% (ENr312) (Figure 3A). None of these are among the worst genomic regions with respect to gaps that we have encountered; the sequences generated for ENCODE pilot project regions ENr231, ENr232, and ENr333 (included in Figure 3A for comparison purposes, but not actually part of the second data set) contain more gaps per Mb (56, 68, and 48, respectively) than any of the other analyzed regions. Similar differences can be seen among the sequences from 21 species in the second data set, with the average total number of gaps ranging from 19 per Mb (galago) to 58 per Mb (platypus), while the fraction of uncaptured gaps varies from 21% (hedgehog) to 50% (elephant and tenrec) (Figure 3B).

The effort required for establishing the order and orientation of sequence contigs--a key part of the sequence-finishing process--directly relates to the number of uncaptured gaps in the sequence assembly. While total gaps per Mb is an important metric to monitor, in practice, it is the presence of two or more uncaptured gaps in a given BAC sequence assembly that presents a significant challenge during sequence finishing. Within the second data set, the fraction of BAC sequence assemblies containing two or more uncaptured gaps ranged from 16% (ENr213) to 47% (ENm003) among the different ENCODE pilot project regions and 12% (vervet) to 60% (platypus) among the different species. BAC sequences from ENCODE regions ENm003, ENm005, ENr111, ENr211 and ENr312 (as well as ENr333 and ENr231) have routinely required extra finishing effort, mostly because over 40% of the sequence assemblies contained two or more uncaptured gaps. Meanwhile, in the case of vervet and squirrel BACs, nearly all sequence assemblies contained fully ordered and oriented contigs, since few of these BAC sequences had two or more uncaptured gaps. In contrast, platypus BAC sequence assemblies had an average of over eight gaps per BAC, with more than 44% of those gaps being uncaptured; this characteristic (in addition to other features of platypus sequence mentioned above) has made the finishing of platypus sequence more challenging than that of any other species' sequence encountered to date.

### Strategies for reducing sequence gaps

In recent years, we have implemented various steps to reduce the number of gaps in our BAC sequence assemblies. For example, in the case of sequence assemblies containing two or more uncaptured gaps, we often will construct fosmid shotgun libraries from the starting BAC DNA, and then generate a modest number (e.g., 96) of sequence read-pairs from fosmid insert-ends. The resulting fosmid clones often capture the DNA residing in previously uncaptured gaps, thereby helping to order and orient the sequence contigs. While effective, such a fosmid-based approach is labor-intensive and expensive; it also introduces delays in finishing a BAC sequence because of the manual nature of fosmid-library construction and subclone-DNA purification. Of note, this approach has not been particularly successful for some species (e.g., platypus). In these cases, the sequence assemblies frequently contain too many contigs that cannot be unequivocally ordered even with fosmid-derived read-pairs (in part because the fosmid insert is too large, with the resulting insert-end reads simply skipping over many contigs and gaps). As an alternative, we found that generating read pairs with a 10- kb insert plasmid library was more productive.

Another approach that we have implemented involves the use of an alternate bacterial host strain for constructing the initial shotgun plasmid library. We reasoned that uncaptured gaps might reflect regions of foreign DNA that are 'poisonous' to the bacterial host, with subclones propagating such DNA in high copy-number plasmids either failing to grow or growing poorly [30-32]. Thus, a host that constrained plasmid copy number might reduce any bacterial growth inhibition, yielding a more uniform distribution of sequence reads across the starting template.

**Figure 4:**
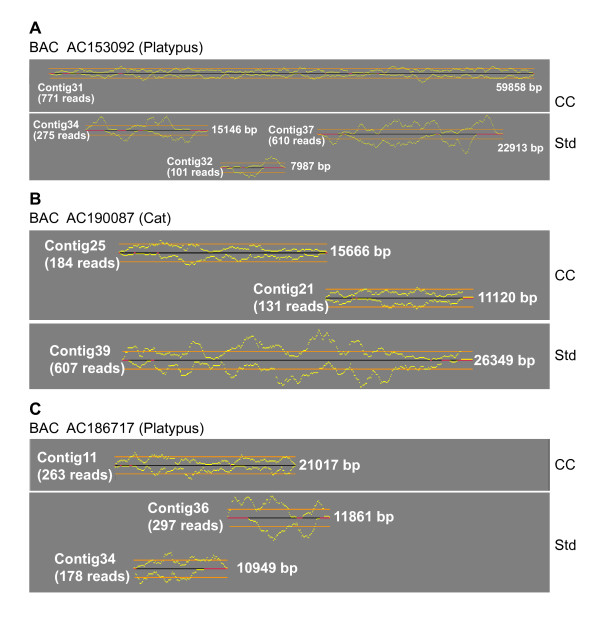
**Variation in the redundancy of sequence reads generated using shotgun libraries prepared with standard and copy-control *E. coli *strains**. A shotgun-subclone library was prepared from each of three BACs [GenBank:AC153092, AC190087, and AC186717] and used to transform either standard DH10B *tonA *(Std) or copy-control EPI400 (CC) *E. coli *strains. From each library, paired forward and reverse sequence reads were then generated from randomly selected subclones to produce assemblies that provided an average of eightfold sequence redundancy. Aligned representative regions of the assemblies that highlight differences in sequence redundancy encountered with the two *E. coli *strains are shown for each BAC. Yellow lines indicate sequence-read redundancies on the upper and lower strands of the indicated sequence contig (black/red line); the horizontal orange lines depict a redundancy value of 10.

To test this idea, we selected three previously generated BAC sequences (produced using our standard bacterial host: *E. coli *DH10B *tonA*). Aliquots of the ligation reactions, which earlier produced the initial shotgun libraries, were used to separately transform competent cells of copy-control *E. coli *strain EPI400 (Epicentre Biotechnologies); BAC sequencing was then performed per our usual routine, after which we compared assemblies from sequence reads obtained with each host strain. Representative results from those comparisons are shown in Figure 4 for a portion (~ 20 to 60 kb) of each of the three BACs. Note the greater uniformity in sequence-read redundancy obtained with the copy-control host strain and the elimination of gaps in two of the BAC sequences.

Based on these initial findings, we performed a similar study with eight additional BACs. Using our standard host strain, the sequence assembly of each of these clones had ≥ 5 uncaptured gaps (Table 5). An additional two BACs [GenBank:AC190000 and GenBank:AC188356] (see Table 5) were included as controls, with their sequence assemblies having 2 and 0 uncaptured gaps, respectively. In most cases, the sequence assemblies generated using the copy-control host cells contained a greatly reduced total number of gaps (especially uncaptured gaps; see Table 5). In several cases, the total number of uncaptured gaps was reduced to <2, thereby eliminating the challenge of ordering and orienting sequence contigs. Interestingly, a significant reduction in the number of gaps per BAC is associated with decreased variation in sequence-read redundancy; we now use this correlation to predict whether a particular BAC sequence assembly with many gaps would benefit from the extra effort of preparing a supplementary shotgun library with copy-control competent cells.

**Table 5:** BAC sequence assemblies generated using standard versus copy-control bacterial host cells.

BAC sequence [GenBank:]	Species	ENCODE Region	Redundancy variation		No. contigs >2 kb		No. uncaptured gaps	
			
			Standard	Copy control	Standard	Copy control	Standard	Copy control
AC190087	Cat	ENm011	++++	-	18	13	6	2
AC153092	Platypus	ENm009	++++	-	15	8	13	3
AC188899	Owl Monkey	ENm006	++++	-	14	9	6	3
AC190076	Owl Monkey	ENm011	++	-	22	15	9	1
AC182792	Shrew	ENr331	++	-	22	11	11	0
AC175233	Shrew	ENr322	++	+	21	22	9	10
AC186717	Platypus	ENm006	++	-	20	10	7	3
AC188506	Rabbit	ENm006	++	+	11	6	5	0
AC190002	Owl Monkey	ENr312	++	++	10	6	5	4
AC187194	Platypus	ENr312	++	+	9	12	7	3
AC172296	Hedgehog	ENr211	+	-	21	13	9	0
AC190000	Owl Monkey	ENm007	+	-	10	6	2	0
AC188356	Dusky Titi	ENm006	+	-	8	13	0	2

Representative BACs whose generated sequences were associated with atypical characteristics (in terms of large numbers of contigs and uncaptured gaps) were tested. Two exceptions [GenBank: AC190000 and AC188356] represent typical sequence assemblies with respect to redundancy of sequence reads, number of contigs, and number of uncaptured gaps. Only three BACs [GenBank: AC190002, AC187194, and AC172296] belong to the second data set (Tables 2 and 3), while the remaining BACs have sequences orthologous to other ENCODE regions. The variation in the redundancy of sequence reads across all contigs was qualitatively assessed as low (-), medium (+), high (++), or very high (++++); see Figure 4 for illustrative examples.

Several points should be made about using such copy-control host strains for shotgun-library construction. First, because there is some reduction in the yields of purified plasmid DNA (and, therefore, sequencing success rates and read lengths) associated with the use of EPI400 cells, we only utilize copy-control host strains in special cases. For example, for species (e.g., platypus) whose initial sequence assemblies regularly have large numbers of gaps, we now routinely use copy-control EPI400 cells during the initial shotgun phase; for other species, these cells are used only after the BAC sequence assembly is reviewed and deemed to require such an approach {with the key determinant being a large variation in sequence-read redundancy (as seen in Figure 4) rather than simply the total number of uncaptured gaps}. Finally, implementing the use of a copy-control host strain is straightforward in a high-throughput sequencing pipeline, making it a convenient and practical option on an as-needed basis. In practice, competent cells derived from the copy-control host strain are simply transformed by the original ligation reaction, eliminating the need to construct an entirely new library in an alternate cloning vector.

### Utilization of 'next-generation' sequencing technologies

Recently, several 'next-generation' DNA sequencing technologies have become commercially available [33-35]. These involve the use of completely different methods for generating shotgun-sequence data, with the resulting sequence reads being considerably shorter than those generated by Sanger-based chemistries (but at a fraction of the per-base cost). We investigated whether data produced with one of these new technologies without cloning bias might complement our standard Sanger-based shotgun sequence reads in a fashion that reduced the problems encountered during sequence finishing.

We selected 12 BACs for this study: 10 whose sequence was previously found to be problematic at the sequence-finishing stage and 2 whose sequence was straightforward to finish (these BACs were derived from 8 different species and 7 different genomic regions). The purified DNA from each BAC was sheared and then ligated with a unique 'indexing' linker. All 12 samples were then mixed together, and the resulting pool then used to make a single shotgun sequencing library using the Genome Analyzer instrument (Illumina) [33]. The resulting paired sequence reads (each being 35 bases in length) were sorted by BAC using the respective indices, with each set of reads then assembled using Velvet [36]; the resulting sequence contigs for each BAC were then assembled with the original Sanger-based shotgun reads using Phrap. Preliminary results showed that the Velvet-derived sequence assemblies differed among the 12 BACs; for example, contig 'N50' size varied from 0.2 to 7 kb, with the 'largest contig' per BAC ranging from 2.8 to 24 kb. Of the contig sequences (totalling 2.35 Mb) that aligned to the Sanger-derived consensus sequences, there were few, if any, mismatches; thus, the quality of the Genome Analyzer-derived assembled sequence was extremely high.

Combining the Sanger- and Genome Analyzer-derived shotgun sequence data simplified some of the sequence-finishing process, but did not solve the most vexing finishing problems. For example, in some cases, the use of Velvet-assembled contigs helped to extend sequence from contig ends, provided complementary strand data, and resolved miscellaneous ambiguities (thereby eliminating the need to generate additional sequence reads during the finishing process). In fact, adding Genome Analyzer-derived data to the Sanger-derived assemblies reduced the total number of contigs for the 10 problematic BACs; for five BACs, a few of the uncaptured gaps were closed. However, new problems sometimes arose with the addition of Velvet-assembled contigs; for example, in several instances, new misassemblies formed where none existed in the Sanger-derived assembly. Most disappointing was the fact that many troublesome gaps in the Sanger-derived assemblies, which had been extremely problematic (and expensive) to close during human-grade sequence finishing, remained unchanged following the incorporation of Velvet-assembled contigs. Although some small contigs were found to partially fill these gaps, much of the missing sequence had no matching Genome Analyzer-derived sequence. These results point out the biases and limitations of both sequencing methods that utilize DNA polymerase.

## Discussion and conclusions

The refinement of sequence assemblies generated from shotgun reads (i.e., sequence finishing) is a multi-step procedure that is influenced by various features of the starting template. Some of these features can present major problems during the sequence-finishing process; the most common being gaps (missing sequence) of various types and characteristics, and sequence misassemblies. Here, we present some representative findings based on our experience in finishing in a standardized fashion the sequence of over 11,000 BACs from 75 different vertebrate species. This unique data resource allows for a rigorous assessment of the requirements for finishing sequence of diverse origins; the current study reveals substantial differences in the effort needed to finish the sequence from different genomic regions and, in particular, from different species.

While our experience provides the opportunity to generate quantitative summaries about the comparative grade-finished assemblies of shotgun sequence from various sources (e.g., Figures 1, 2 and 3), it also allows general qualitative conclusions to be drawn. For example, we have found that assembled shotgun sequence from: (1) rabbit and platypus sequences contain a higher than average number of SINEs and LINEs in and around regions associated with gaps, making the design of unique PCR primers for capturing the missing DNA impossible; (2) owl monkey, colobus monkey and tenrec sequence assemblies are often associated with regions of up to 2 kb that are biased with respect to the strand from which the shotgun reads were derived; (3) shrew and platypus are associated with a high frequency of large, uncaptured gaps; (4) dog and shrew are associated with gaps whose underlying sequence has a high GC content; and (5) baboon is associated with frequent misassemblies. Especially troublesome was the finding that hedgehog BACs are often associated with large deletions (50-100 kb) that occur during culturing of the clones prior to DNA extraction. In addition to the conclusions derived from analyzing the data sets reported here, we have gained considerable experience finishing sequences from other vertebrates. Based on this experience, we have found that: (1) little brown bat sequence assemblies are associated with numerous problematic dinucleotide repeats and a high frequency of uncaptured gaps whose underlying sequence has a high GC content; (2) opossum sequences are associated with large stretches of dinucleotide repeats at contig ends, often causing non-contiguous sequences to be incorrectly joined during assembly; and (3) echidna assemblies are associated with a high frequency of large, uncaptured gaps and with numerous problematic dinucleotide repeats. Interestingly, such features of the assembled sequence actually point to characteristic differences in genomic architecture among species.

The insights gained from examining the underlying causes of sequence-finishing problems have allowed us to develop approaches to improve the finishing process, in some cases through proactive steps implemented in a species-specific fashion. For example, changing the host strain used for propagating shotgun subclones can improve the representation of certain sequences and reduce problems associated with sequence gaps. The use of different assembly programs can in some cases help to reduce the frequency and severity of misassembled sequences. Finally, relevant to recent technological advances, supplementing Sanger-derived shotgun reads with sequence data generated with a 'next-generation' DNA sequencing platform (e.g., an Illumina Genome Analyzer) can recover some of the sequence that is otherwise missing in initial assemblies. We are investigating further alternative approaches for employing these new platforms in generating sequence data that are more complete, reducing the effort required for finishing (unpublished data).

However, our experience also confirms the continued labor-intensive nature of sequence finishing and the absence of any 'magic bullet' that resolves all major sequence-finishing problems. Some of the most challenging sequences to finish are from species for which a draft genome sequence has already been generated and currently sits at a draft stage, such as for dog [37] and platypus [38]. In fact, draft whole-genome sequences of various levels of completion have been generated for most of the more than 30 species studied here [8,9]. Our findings may provide important insights that will aid future efforts to refine those draft sequence assemblies. If high-quality finished sequences are eventually to be produced for all of these vertebrate genomes, then continued vigilance about understanding the general and species-specific challenges associated with sequence finishing will be needed, as will methodological advances to further reduce the cost and to improve the efficiency of the sequence-finishing process.

## Methods

### Sequence generation

Purified BAC DNA was sheared and 'shotgun subcloned' into plasmids containing 3- to 5- kb inserts using *E. coli *DH10B *tonA *host cells (Invitrogen). Plasmid DNA was purified from individual subclones by an automated magnetic bead process (Agencourt Bioscience). Paired sequence reads were generated from randomly selected subclones by universal forward- and reverse-primed fluorescent dideoxy sequencing reactions [1,2] using AB3730*xl *sequencing instruments (Applied Biosystems). Typically, 2,500-2,800 sequence reads were generated for each BAC (which averaged ~ 165 kb in size), with the resulting Phrap-based [25,26] sequence assembly consistently providing at least eight-fold redundancy of the starting BAC.

### Sequence finishing

Sequence assemblies were refined to produce comparative-grade finished sequence; this process, described in detail by Blakesley *et al*. [5], including a summary of the step-by-step procedures within the Supplementary Materials of that paper, mostly involved a series of computational procedures that yields an ordered and oriented map of all sequence contigs greater than 2 kb in size. That map was routinely verified with independent data, including sequence derived from overlapping BACs, alignments with orthologous sequence from another species, and/or results of PCR-based experiments to confirm contig adjacency. A subset of the BACs was further finished to the 'human-grade' standards established for sequencing the human genome [4,39]. The GenBank accession numbers for all sequenced BACs analyzed here are available on request.

All sequencing reactions for the shotgun phase and most reactions for the finishing phase employed Big-Dye Terminator chemistry (Applied Biosystems) as described by the manufacturer. Certain other sequence finishing reactions used alternative chemistries, where dGTP BigDye Terminator Mix (Applied Biosystems) or an 80:20 mixture of BigDye:dGTP Big Dye substituted for BigDye Terminator Mix in the sequencing reaction mixture. In certain cases, one-tenth volume of SeqRxA (Invitrogen) was added to the reaction.

## Authors' contributions

RWB, NFH, JCM, BM, GGB, and EDG conceived the study. NFH led the bioinformatics analyses, with contributions from JCM, BM, GGB, and RWB. JG, BBB, SYB, HC, PH, S-LH, KS, SS, JLV, PJT and members of the NISC Comparative Sequencing Program generated the data and performed some of the bioinformatic analyses. RWB, NFH, and EDG wrote the manuscript.
